# Non-Volatile Bioactive Properties of Mushroom Extracts (*Agaricus bisporus* and *Pleurotus ostreatus*)

**DOI:** 10.3390/molecules31091397

**Published:** 2026-04-23

**Authors:** Agnieszka Nowak, Małgorzata Piotrowska, Karolina Przydacz, Agata Czyżowska, Katarzyna Rajkowska, Katarzyna Dybka-Stępień, Anna Koziróg, Anna Otlewska, Grażyna Budryn, Anna Kołczyk

**Affiliations:** 1 Institute of Fermentation Technology and Microbiology, Lodz University of Technology, Wolczanska 171/173, 90-530 Lodz, Poland; karolina.przydacz@gmail.com (K.P.); agata.czyzowska@p.lodz.pl (A.C.); katarzyna.rajkowska@p.lodz.pl (K.R.); katarzyna.dybka@p.lodz.pl (K.D.-S.); anna.kozirog@p.lodz.pl (A.K.); anna.otlewska@p.lodz.pl (A.O.); 2 Institute of Food Technology and Analysis, Faculty of Biotechnology and Food Sciences, Lodz University of Technology, Stefanowskiego 2-22, 90-537 Lodz, Poland; grazyna.budryn@p.lodz.pl; 3 FoodHub S.A., Wielunska 2, 97-438 Rusiec, Poland; anna.kolczyk@foodhub.com.pl

**Keywords:** mushroom extracts, non-volatile components, broth, zero-waste food

## Abstract

Sustainable food processing and zero-waste management of by-products require the search for natural functional ingredients that can be used in finished food products. Edible mushrooms are a rich source of non-volatile bioactive compounds, but their functionality in complex food matrices remains poorly understood. The aim of the study was to compare the profiles of bioactive non-volatile compounds in *Pleurotus ostreatus* and *Agaricus bisporus* extracts obtained by the ultrasonic and combined (shaking + ultrasound) methods and to assess the possibility of using the selected extract in zero-waste broths. The content of DNA, polyphenols, nucleosides and nucleotides, and low-molecular-weight metabolites, as well as the antioxidant activity of the extracts and broths, was assessed. Significant species and technological differences in extraction efficiency were demonstrated, with *A. bisporus* extracts obtained using the combined method characterized by the highest content of compounds with reducing potential. Adding 2% *A. bisporus* extract to the broth increased the reduction potential (FRAP) and selectively altered the nucleoside and polyphenol profile, without significantly affecting key umami nucleotides. The results provide preliminary evidence suggesting that mushroom extracts could be considered as functional ingredients in zero-waste products, with the potential to enhance their bioactive properties.

## 1. Introduction

The modern food industry faces the growing challenge of reducing raw material losses and effectively managing by-products, in line with global sustainable development strategies and the zero-waste concept. Globally, food waste is valued at nearly $1 trillion per year according to the FAO, with the United States alone losing about $161 billion annually at retail and consumer levels. In the European Union, food waste is estimated at EUR 800 billion per year [1,2]. A significant portion of food waste concerns both plant-based raw materials, such as vegetables rejected due to visual defects, and animal-based products, including residues from mechanical meat separation (MSM) processes. In this context, broths and stocks offer practical possibilities for the management of by-products, constituting a complex matrix in which non-volatile components can contribute to shaping the product properties [3,4].

Edible mushrooms have long been recognized as a valuable dietary component, and in recent years, they have gained increasing interest as a source of bioactive compounds with functional potential. Mushroom fruiting bodies contain a wide range of low-molecular-weight primary and secondary metabolites, including polyphenols, nucleosides and nucleotides, polyols, and polysaccharides, which exhibit antioxidant, immunomodulatory, and protective activity [5,6,7,8]. Among the species commonly available on the market, *Agaricus bisporus* and *Pleurotus ostreatus* deserve special attention due to differences in cell wall structure, which determine the efficiency of bioactive compound extraction [9].

Previous studies have focused mainly on characterizing the chemical composition of raw or dried mushroom fruiting bodies and their extracts, often analyzed in isolation from actual food matrices. In the available literature, you can find information about research on the addition of edible mushroom extracts (aqueous, ethanolic) to meat, fish, dairy, and cereal products, and beverages as fortifiers aimed at enhancing the antioxidant and anti-inflammatory effects, as well as preservatives, with a lesser impact on taste/consistency [10,11,12,13]. Available research indicates that mushroom powders and extracts are multifunctional ingredients that improve taste and nutritional value, and serve as a source of fiber and antioxidants in soups and instant mixes [12,13,14,15]. In technological practice, it is crucial not only to maximize the recovery of individual compounds but also to balance improvements in functional properties with an acceptable sensory profile of the product.

An important factor determining the composition and biological activity of mushroom extracts is the extraction method used. Intensive physical methods such as ultrasound, combined with mechanical agitation, effectively disrupt cellular structures and release compounds bound to the cell wall matrix. At the same time, these processes can selectively shape the profile of metabolites extracted, promoting the release of specific classes of compounds with reducing and antioxidant potential [16,17,18]. In this approach, the importance of analyzing the so-called “non-volatile bioactive compound profile” is increasingly emphasized, instead of striving for a full, comprehensive characterization of all flavor carriers, which in the case of complex food matrices can be methodologically difficult and interpretatively ambiguous. From an application perspective, it is particularly important to distinguish between flavor intensification and improvements in product fullness and sensory stability. Many non-volatile mushroom components, such as nucleosides, nucleotides other than classic 5′-monophosphates, and selected phenolic compounds, do not lead to a dramatic increase in flavor intensity, but rather contribute to its complexity, persistence, and body. Multidimensional sensory–chemical studies show that, in addition to volatile substances, non-volatile compounds (sugars, acids, amino acids, nucleotides) can shape the intensity of taste and influence consumer preferences for different mushroom species [19,20]. This type of interaction is desirable in zero-waste products, where the goal is to harmonize and stabilize the sensory profile rather than to aggressively modify it.

Despite growing interest in mushrooms as functional ingredients, there is still a lack of research that consistently compares different mushroom species and extraction methods, and assesses the effect of the selected extract on the functional properties of the finished food product. In particular, few studies examine the use of mushroom extracts in broths prepared using by-products, which are an essential element of a zero-waste strategy. Mushroom extracts are currently used in the dairy, meat, bakery, starchy foods, and beverage industries. In dairy products, they are predominantly antioxidants and antimicrobials, with microencapsulation allowing for stabilization and regulated release upon storage [11]. They operate as natural preservatives and fat, salt, and phosphate alternatives in meat products and their counterparts, increasing shelf life and improving health profiles while retaining sensory properties [12,13]. They raise fiber, protein, and antioxidant levels in cereals and beverages while also influencing texture, color, and bioavailability of bioactive substances [21,22]. This gap particularly concerns assessing whether adding mushroom extract can improve the product’s antioxidant potential without disrupting its basic flavor profile. Therefore, the aim of this study was to compare the profiles of non-volatile bioactive compounds in extracts from *Pleurotus ostreatus* and *Agaricus bisporus* obtained using different extraction methods and to assess the potential use of the selected mushroom extract as a functional ingredient in zero-waste broths. This research aimed to provide knowledge useful from a food technology perspective, combining functional, sensory, and sustainable processing aspects.

## 2. Results and Discussion

Table 1 presents a comparison of the extraction efficiency of biologically active compounds from *Pleurotus ostreatus* and *Agaricus bisporus* fruiting bodies using ultrasonic disintegration (U) and the combined method: shaking and ultrasound (S+U). The content of single-stranded (ssDNA) and double-stranded DNA (dsDNA), total polyphenolic compound content (TPC), and antioxidant activity, expressed as the ability to neutralize 2,2′-azinobis-3-ethylbenzothiazoline-6-sulfonate (ABTS) and 2,2-diphenyl-1-picrylhydrazyl (DPPH) free radicals, was assessed. The obtained results clearly indicate that both the mushroom species and the extraction method used are important for the efficiency of obtaining biologically active compounds. The differences between *Pleurotus ostreatus* and *Agaricus bisporus* may result from differences in the structure of the cell wall and the chemical composition and degree of cross-linking of structural polysaccharides. The significant increase in ssDNA and dsDNA content in *A. bisporus* extracts obtained by the S+U method suggests that the combination of shaking with ultrasound more effectively disrupts the cellular structure of this fungal species. The high total nucleic fraction content in the *A. bisporus* extract obtained by the S+U method is noteworthy. This is likely due to both the effective release and partial denaturation of nucleic acids during ultrasonication and shaking, as well as the presence of co-extracted compounds (e.g., RNA, nucleotides, and nucleosides), which may overestimate the spectrophotometric measurement result. *A. bisporus* cell walls, rich in chitin and β-glucans, may require more intense mechanical action to enable the release of genetic material and low-molecular-weight compounds. In contrast, in *P. ostreatus*, the improvement in DNA extraction efficiency was moderate, suggesting a greater susceptibility of this species to ultrasonication. Beta-glucan and chitin are the primary structural polysaccharides in the cell walls of edible mushrooms. Their proportions and structural arrangements influence not only the mushrooms’ texture and biological activity but also their potential applications in food, health, and biotechnology. In both species, *P. ostreatus* and *A. bisporus* β-glucans (mainly β-1,3/1,6 linkages) form the bulk of the cell wall matrix, with chitin providing additional rigidity. In *A. bisporus*, β-glucan is often tightly associated with chitin, making extraction more challenging [23,24,25].

The lack of significant differences in TPC content between extraction methods for *P. ostreatus* may indicate that the polyphenols present in this species are readily available and do not require intensive disintegration methods. However, the significant increase in TPC in *A. bisporus* extracts obtained by the S+U method confirms that polyphenols of this species may be more strongly bound to the cell matrix, e.g., through interactions with polysaccharides or proteins. Polyphenols in both species are often associated with the cell wall matrix. This association can be non-covalent (hydrogen bonding, hydrophobic interactions) or, less commonly, covalent, affecting their solubility and bioavailability [24,26].

The antioxidant activity of extracts did not always correlate directly with polyphenol content. In the case of *P. ostreatus*, the higher ABTS radical scavenging capacity in extracts obtained by the U method, despite a comparable TPC, suggests the involvement of other antioxidant compounds, such as polysaccharides, peptides, or phenolic compounds with high individual activity. In the case of *A. bisporus*, the significant improvement in antioxidant activity measured by the ABTS method using S+U may be directly related to increased polyphenol extraction. In summary, combining shaking with ultrasound is an effective strategy for intensifying the extraction of biologically active compounds, especially from *A. bisporus*. These results emphasize the importance of selecting the extraction method based on the mushroom species and the type of target bioactive compounds.

Polyphenols are important bioactive compounds in edible mushrooms, contributing to their antioxidant, antimicrobial, and health-promoting properties. Both button mushrooms and oyster mushrooms are recognized sources of polyphenols. The analysis of the phenolic profile of *Pleurotus ostreatus* and *Agaricus bisporus* extracts showed significant differences, both quantitative and qualitative, between the tested species and the extraction methods used (Table 2). Hydroxybenzoic acids, mainly gallic acid (GAE) and protocatechuic acid (PCA), dominated in *Pleurotus ostreatus* extracts, regardless of the extraction method. The concentration of GAE was significantly higher after ultrasonic extraction than by the S+U method, while PCA reached significantly higher concentrations after the combined method, which indicates the differential availability of compounds of the same phenolic group. In *Agaricus bisporus*, the phenolic profile was dominated by vanillic acid (VA) and caffeic acid (CA). The highest VA concentration was observed in extracts obtained by the ultrasonic method, whereas the S+U method resulted in a significant reduction. Chlorogenic acid (CQA) was present at very low concentrations in all samples, whereas ferulic acid (FA) was detected only in *P. ostreatus* extracts, where its content was significantly higher after the S+U extraction method. Flavonoids, represented by quercetin glucoside (QG), had a limited share in *P. ostreatus* extracts, whereas in *A. bisporus*, they were detectable only after using the S+U extraction method. In summary, the hydroxybenzoic acids dominated in both mushroom species. *P. ostreatus* is characterized by a predominance of gallic and protocatechuic acids, while *A. bisporus* is characterized by a predominance of vanillic acid, and the extraction method modifies the proportions of individual compounds within these groups without altering the overall species trend. Available literature data clearly indicate that the relative proportions of hydroxybenzoic acids, hydroxycinnamic acids, and flavonoids in edible mushrooms are not constant and are strongly dependent on species, cultivar, and cultivation conditions, resulting in significant variability in reported phenolic profiles. Numerous studies emphasize the lack of a single universal pattern of phenolic class dominance, which complicates direct quantitative comparisons between studies and requires cautious interpretation of results [27,28]. Research conducted by Machado-Carvalho et al. (2023) showed that *A. bisporus* extracts are characterized by a higher content of polyphenolic compounds than *P. ostreatus*, which also translates into higher antioxidant activity [29].

Studies on *Pleurotus ostreatus* indicate that the phenolic profile of this species may include significant contributions from hydroxybenzoic acids and hydroxycinnamic acids, with a relatively smaller but variable contribution from flavonoids. Matkovits et al. (2024) demonstrated that the differences between *P. ostreatus* cultivars are so pronounced that the proportions between phenolic classes, rather than the presence of individual compounds, are the key factor enabling their effective classification [30]. Comparative and review studies of edible mushrooms indicate that phenolic acids—both hydroxybenzoic and hydroxycinnamic acids—are the most prevalent group of polyphenols, while flavonoids are typically present in smaller amounts, although they can significantly influence the biological properties of extracts [28]. This is consistent with our research results.

The profile of low-molecular-weight primary metabolites determined in the extracts of *Agaricus bisporus* and *Pleurotus ostreatus* included organic acids and soluble carbohydrates, including sugar alcohols (polyols) (Table 3). Among the carbohydrates identified, mannitol was the dominant compound in *A. bisporus* extracts, regardless of the extraction method used. The literature clearly indicates that this polyol is the main form of carbon storage in this species, in contrast to *P. ostreatus*, where trehalose is more commonly the storage form [31]. The studies by Sławińska et al. (2021) showed that mannitol is the most abundant free sugar alcohol in the fruiting bodies of *A. bisporus* (5.4–7.6 mg/g d.m.), while its content in *P. ostreatus* is several times lower, which fully corresponds to the results of our study [32]. The high mannitol content in *A. bisporus* has physiological significance, as this compound functions not only as a carbon reservoir but also as a regulator of osmotic and redox balance in fungal cells. Its accumulation is closely linked to intense energy metabolism and the activity of the tricarboxylic acid (TCA) cycle, which is reflected in the simultaneous presence of significant amounts of organic acids, such as malic and acetic acids. It is also important that free sugars and polyols, alongside organic acids, are considered non-volatile compounds that contribute to the sensory profile of mushrooms. As Sławińska et al. (2021) emphasize, mannitol, glucose, and trehalose contribute to the mild sweetness and full flavor, and their proportions are characteristic of individual species [32]. It is also worth noting that the literature on mushroom processing and valorization emphasizes the high sensitivity of low-molecular-weight metabolite fractions to technological conditions. Bermúdez-Gómez et al. (2024) demonstrated that physical processes, such as drying or mechanical tissue damage, can significantly modulate the chemical composition of mushrooms, affecting the availability of soluble carbohydrates and organic compounds [33]. In this context, the effect of the extraction method on the recovery of mannitol and organic acids, observed in this study, is consistent with a broader trend reported in the literature.

5′-phosphorylated ribonucleotides play a significant role in shaping food flavor, primarily by enhancing the umami sensation. Of these compounds, 5′-guanosine monophosphate (5′-GMP) and 5′-inosine monophosphate (5′-IMP) are of greatest sensory importance. While these compounds have only a weak flavor on their own, they act synergistically with glutamic acid, significantly increasing the intensity of the umami sensation. This mechanism involves stabilizing the umami receptors (T1R1/T1R3), leading to stronger and longer-lasting flavor perception [34]. The nucleoside and ribonucleotide profiles in *Pleurotus ostreatus* and *Agaricus bisporus* extracts showed clear species differentiation and a significant effect of the extraction method used. In all analyzed samples, the fraction designated as “other nucleotides and nucleosides” dominated, indicating that a significant portion of purine and pyrimidine compounds occurred in forms other than classical 5′-monophosphates (Table 4). It is consistent with previous reports on the composition of non-volatile flavor components in edible mushrooms [20,35].

It should be emphasized, however, that this fraction does not have to include only free nucleosides or nucleotide degradation products, but may also contain ribonucleotides esterified in the 2′ and/or 3′ positions, which are naturally present in fungal tissues and do not show a taste activity comparable to 5′-phosphorylated ribonucleotides. Ranogajec et al. (2010) point out that standard HPLC methods used to determine nucleosides and nucleotides in fungi do not always allow for a clear distinction between 2′-, 3′-, and 5′-phosphate isomers, which may lead to their co-elution in a single analytical fraction [35]. In the *P. ostreatus* extracts, the presence of 5′-CMP and 5′-TMP was determined, whereas neither 5′-AMP nor purine nucleotides (5′-GMP and 5′-IMP) were detected. In the *A. bisporus* extracts, the dominant nucleotide was 5′-UMP, and its content increased significantly after the combined method (S+U). This variation is consistent with the results of Tsai et al., who showed that the composition and amount of 5′-nucleotides in *A. bisporus* strongly depend on the developmental stage of the fruiting bodies and the processing conditions of the raw material [36]. Of particular note is the absence of 5′-GMP and 5′-IMP in any of the samples. This is important from a sensory perspective because, as mentioned earlier, these nucleotides are key umami flavor enhancers that work synergistically with glutamic acid. Studies on the molecular mechanism of umami perception clearly indicate that only 5′-phosphorylated ribonucleotides, such as GMP and IMP, stabilize the active conformation of the T1R1/T1R3 receptor, while the 2′- and 3′-phosphate isomers do not exhibit such activity [34]. The absence of these nucleotides in the determined fraction does not necessarily mean a complete absence of purine compounds, but may indicate that they occurred in isomeric forms or were partially transformed during extraction. Yin et al. (2019) demonstrated that in *Pleurotus* fungi, water and heat treatments promote the degradation of 5′-nucleotides to nucleosides or phosphate isomers, leading to a decrease in their directly determined content [20]. The effect of the extraction method was visible in both fungal species—the S+U method led to a significant increase in the content of the “other nucleosides and nucleotides” fraction. This phenomenon can be interpreted as resulting from more effective release of compounds associated with RNA and possible isomerization of the phosphate position during intensive extraction, as previously described in studies on the stability of ribonucleotides in biological matrices.

The results of studies on the extracts obtained confirmed that extraction efficiency and the composition of biologically active compounds strongly depend on the mushroom species and the extraction method used. The combined method (S+U) was particularly effective for *Agaricus bisporus*, which has a more compact cell wall structure. *A. bisporus* S+U extracts were characterized by the highest polyphenol content, high antioxidant activity, and the dominance of mannitol and organic acids, which together have the potential to shape the full flavor. *Pleurotus ostreatus* showed less dependence on the extraction method. The nucleoside profile was dominated by forms other than 5′-monophosphates, indicating that the sensory qualities of the extracts are mainly due to the synergy of non-volatile metabolites. In summary, *A. bisporus* extract obtained by the S+U method is the most promising addition to zero-waste broths, combining sensory, functional, and technological values. Accordingly, broths obtained from poultry carcasses remaining after MSM production and from out-of-grade vegetables were enriched with *Agaricus bisporus* extract.

**Figure 1:**
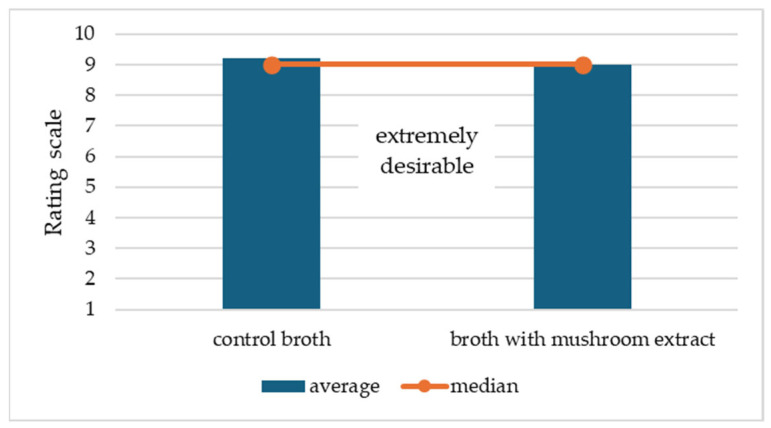
Consumer-based organoleptic evaluation of control and mushroom extract-enriched broth.

The results presented in Table 5 show that the addition of 2% *Agaricus bisporus* extract selectively influenced the profile of non-volatile bioactive compounds in the broth, without causing significant changes in its basic flavor profile. In terms of the determined 5′-phosphorylated ribonucleotides, no significant differences were found in the content of 5′-CMP, 5′-TMP, or key umami nucleotides (5′-GMP and 5′-IMP) between the control and enriched broth, which indicates that the intensity of the umami taste, determined mainly by the meat raw material, was not significantly modified. At the same time, a significant increase in the content of 5′-AMP and the fraction of “other nucleosides and nucleotides” was observed in the enriched broth, which is consistent with the profile of the *A. bisporus* extract and suggests an enhancement of the fullness and persistence of the taste sensation, rather than its intensification. The addition of the extract also significantly affected the polyphenol profile. The enriched broth showed a significant increase in the content of hydroxybenzoic acid derivatives, while simultaneously reducing the content of hydroxycinnamic acid derivatives, reflecting the characteristic phenolic composition of *A. bisporus* and the varying stability of individual groups of compounds in an aqueous environment. Flavonoids were present in only trace amounts in both variants, confirming their marginal importance in this matrix. The changes in chemical composition were reflected in the results of antioxidant activity assays. The lack of significant differences in ABTS radical scavenging capacity indicates that the total free radical scavenging potential remained similar. However, the significant increase in FRAP values in the enriched broth indicates an increased reduction potential, which may be attributed to the presence of electron-donating compounds such as polyphenols, nucleosides, and nucleotides from the mushroom extract. Figure 1 presents the results of the consumer-based organoleptic evaluation of the control broth and the broth enriched with yeast extract, taking into account attributes such as taste, aroma, appearance, and consistency. Adding the extract did not lead to major changes in overall acceptability, suggesting that the product’s sensory profile remained generally comparable to the control. The results obtained confirm that the use of 2% *A. bisporus* extract improves the functional properties of the broth while maintaining its sensory balance. It should be noted that the use of only one concentration of *Agaricus bisporus* extract limits the ability to assess a dose–response relationship. Therefore, the observed effects cannot be generalized to other concentrations, and further studies involving a wider range of extract concentrations are necessary to confirm and extend these findings.

## 3. Materials and Methods

### 3.1. Extraction

The extracts were prepared using three batches of fresh button mushrooms (*Agaricus bisporus*) and oyster mushrooms (*Pleurotus ostreatus*) purchased from a local store (country of origin: Poland, producer: EUROFUNGI, Dobroń, Poland and HAJDUK GROUPE, Lipinki, Poland).

The mushrooms were washed in running water and drained on a paper towel, then homogenized using a laboratory homogenizer Bio-Gen Series PRO200 (Pro Scientific; Oxford, CT, USA), resulting in a heterogeneous particle size distribution. A defined particle size was not determined; however, this approach reflects typical processing conditions used in practical applications. Extraction was carried out with water as eluent in a mushroom:water 1:1 (*w*/*v*) ratio using two methods: (1) ultrasonic disintegration at a frequency of 40 kHz, at a temperature of 20 °C for 60 min in an ultrasonic bath Sonic 5 (Polsonic Palczynski Partnership, Warsaw, Poland); (2) ultrasonic disintegration (as above), followed by shaking for 60 min at 150 rpm in orbital shaker Unimax 1010 (Heidolph Scientific Products GmbH, Schwabach, Germany). The extraction conditions were selected based on preliminary experiments (unpublished data). The extracts were filtered through Whatman No. 4 filter paper and were centrifuged at 3000 G for 20 min (BR4i multifunction centrifuge, Thermo Fisher Scientific Inc, Waltham, MA, USA). The obtained supernatants were frozen at −20 °C and used for analysis. Due to the small number of batches analyzed (n = 3), the reproducibility of the results of all extract analyses was described using standard deviation as a basic measure of data dispersion.

### 3.2. Determination of Nucleic Acid Concentrations in Mushroom Extracts

To compare extraction efficiency methods, nucleic acid concentrations were determined using a NanoPhotometer^®^ Pearl nanospectrophotometer (Implen GmbH, Munchen, Germany). Nucleic acid absorbance was measured at 260 nm. A total of 2 μL of extract was applied to the detection window. A lid with a dilution factor of 10 was used. In these systems, other UV-absorbing compounds, including RNA, free nucleotides, and nucleosides, are co-extracted and can contribute to the signal measured at this wavelength. The ssDNA (single-strand DNA) and dsDNA (double-strand DNA) concentrations were estimated using instrument-specific conversion factors and expressed in μg/mL. This approach allows rapid assessment of nucleic acid content, with the resulting values interpreted as relative indicators of the overall nucleic acid fraction.

### 3.3. Estimation of Total Polyphenol Compounds in Mushroom Extracts

The content of polyphenolic compounds in the extracts was determined by the Folin–Ciocalteau method [37]. The reaction mixture was prepared by mixing 0.1 mL of the extract with 0.2 mL of the Folin–Ciocalteau reagent (Chempur, Piekary Sl., Piekary Śląskie, Poland), 2.0 mL of sodium carbonate solution (20% *w*/*v*), and 1 mL of water. Samples were mixed and incubated for 1 h in the dark at room temperature. Absorbance was determined at 765 nm (V1200 spectrophotometer, VWR International Ltd., Gdansk, Poland) against a reference sample (water instead of the test sample). A calibration curve was constructed with varying concentrations (5–170 μg/mL) of gallic acid as standard. The results were expressed as mg of gallic acid equivalents (GAE) per mL extract.

### 3.4. DPPH (2,2-diphenyl-1-picrylhydrazyl) Radical Scavenging Assay

The assessment of antioxidant activity was carried out using a slightly modified method according to [38]. Initially, a solution of 24 mg/L DPPH (2,2-diphenyl-1-picrylhydrazyl, Sigma Aldrich, St. Louis, MO, USA) in 80% (*v*/*v*) ethanol was prepared, followed by a reaction mixture containing 50 μL of mushroom extract and 1.95 mL of the DPPH solution. Water was added to the reference sample instead of the extract. After 30 min of incubation at room temperature in the dark, absorbance at 515 nm was measured (V1200 spectrophotometer, VWR International Ltd., Gdansk, Poland). The percentage of DPPH free radical scavenging was determined using the following formula:

$$\%=\frac{A_{0}-A_{1}}{A_{0}}\;\cdot 100$$

*A*_0_ is the absorbance of the reference sample, and *A*_1_ is the absorbance of the sample.

### 3.5. ABTS Radical Scavenging Assay

The ABTS radical was prepared by mixing 19.8 mg of ABTS (2,2′-azinobis-(3-ethylbenzothiazoline-6-sulfonate), Sigma-Aldrich, St. Louis, MO, USA) reagent, 3.3 mg of K_2_S_2_O_8_, and 5 mL of distilled water [39]. The solution was incubated at room temperature, protected from light, for 16 h. After this time, 2 mL of the solution was added to 138 mL of distilled water, and the absorbance was determined at 734 nm. The solution was then diluted to approximately 0.700. A reaction mixture containing 30 μL of mushroom extract and 3.0 mL of the ABTS solution was then prepared. Water was added to the reference sample instead of the extract. After 15 min of incubation at room temperature in the dark, absorbance at 734 nm was measured (V1200 spectrophotometer, VWR International Ltd., Gdansk, Poland). The percentage of ABTS free radical removal was determined as described above. Antioxidant activity was additionally expressed as Trolox (6-hydroxy-2,5,7,8-tetramethylchromane-2-carboxylic acid, Sigma-Aldrich, St. Louis, MO, USA) equivalent [mg/L] based on the standard curve.

### 3.6. Assessment of Antioxidant Activity by the FRAP Method

The FRAP reagent was prepared by mixing a TPTZ (2,4,6-tris(2-pyridyl)-1,3,5-triazine, Sigma-Aldrich, St. Louis, MO, USA) solution in 40 mM HCl, an FeCl_3_ × 6 H_2_O solution (5.4 g/L), and a phosphate buffer at pH 3.6 in proportions of 1:1:100. Next, a reaction mixture was prepared containing 50 μL of broth and 1.95 mL of FRAP reagent [40]. After incubation of the samples for 30 min at 37 °C in the dark, the absorbance was determined at 593 nm (V1200 spectrophotometer, VWR International Ltd., Gdansk, Poland) against a reference sample (water instead of the test sample). Antioxidant activity was expressed as FeSO_4_ × 7 H_2_O [mg/L] based on the standard curve.

### 3.7. Determination of Polyphenols in Mushroom Extracts by HPLC Method

Mushroom extracts were filtered using 0.45 µm membrane filters. Analysis was performed by liquid chromatography using a Finnigan^TM^ Surveyor Plus^TM^ chromatograph (Thermo Separation Products, Riviera Beach, FL, USA) equipped with an autosampler and a photodiode array detector (Finnigan Surveyor PDA Plus Detector) and controlled by ChromQuest 5.0 software (Thermo Fisher Scientific Inc., Waltham, MA, USA). Separation was achieved using a Spherisorb ODS2 column (250 × 4.6 mm × 5 μm packing) (Waters Corp., Milford, MA, USA), protected by a precolumn of the same material. The sample volume was 50 μL, the flow rate was 0.8 mL/min, and the separation time was 60 min. The mobile phase consisted of 5% formic acid (phase A) and 95% acetonitrile (phase B). Samples were eluted using a gradient method. The compounds were identified by comparing the retention times of the standard compounds with those of the compounds in the extracts. Polyphenolic compounds were quantified based on peak areas using calibration curves for GAE—gallic acid (280 nm), PCA—protocatechuic acid (280 nm), VA—vanillic acid (280 nm), CQA—chlorogenic acid (320 nm), CA—caffeic acid (320 nm), FA—ferulic acid (320 nm), and QG—quercetin glucoside (360 nm). The concentration of polyphenolic compounds was expressed in mg per liter of mushroom extracts [mg/L].

### 3.8. Determination of Selected Organic Compounds in Mushroom Extracts by HPLC Method

Estimation of selected organic acids, sugars, and sugar alcohols in mushroom extracts was performed using the HPLC equipment described above. Separation was achieved using an Aminex HPX-87H column (300 × 7.8 mm packing) (Bio-Rad Laboratories Inc., Hercules, CA, USA). The sample injection volume was 10 μL, the flow rate was 0.6 mL/min, and the separation time was 40 min at 30 °C. The mobile phase consisted of 5 mM H_2_SO_4_. Compounds were identified by comparing the retention times of standard compounds to those of the compounds in the extracts. Organic acids were quantified using calibration curves for malic, acetic, and lactic acids. Sugars were quantified using calibration curves for glucose and maltose. Mannitol, as one of the main sugar alcohols, was also quantified using a calibration curve. The concentration of organic compounds was expressed as mg per liter of mushroom extracts [mg/L].

### 3.9. Estimation of Nucleoside Content in Mushroom Extracts by HPLC Method

Determination of nucleoside 5′-phosphates in mushroom extracts was performed by the HPLC method [33] using the equipment described in 2.6 point. Separation was achieved using a Spherisorb ODS2 column (250 × 4.6 mm × 5 μm packing) (Waters Corp., Milford, MA, USA), protected by a precolumn of the same material. The sample injection volume was 50 μL, and the flow rate was 0.6 mL/min. The mobile phase consisted of 10 mM KH_2_PO_4_, pH 5.6 (solvent A) and 100% methanol (solvent B). A gradient elution program was applied as follows: 0–25 min linearly increased from 0% to 20% B and then decreased to 0% B in 1 min. The composition was held at 0% B for a further 14 min for reequilibration, giving a total run time of 40 min.

Nucleoside 5′-phosphates were quantified according to calibration curves prepared for 5′AMP, 5′CMP, 5′GMP, 5′UMP, 5′IMP, and 5′TMP. Additionally, based on spectral analysis, compounds belonging to the nucleoside and nucleotide groups were identified. The concentration of nucleosides and nucleotides in the extract was expressed as mg/L of 5′AMP.

### 3.10. Preparing a Zero-Waste Chicken Broth with Mushroom Extract

The broth was prepared using chicken carcasses after separating the cooking portions and high-quality vegetable waste: carrots, celery, parsley, and onion. The proportions of carcasses/water/carrots/celery/parsley/onion were 1:2:0.2:0.2:0.2:0.1. Cooking was carried out in a pressure vessel for 3 h at a temperature of 122 °C and a pressure of 0.2 MPa. After cooking was completed and the temperature was lowered below 70 °C, a selected *Agaricus bisporus* extract (U+S) was added in the amount of 2% *v*/*v*. The reference sample was the basic broth without the addition of extract. The antioxidant activity in the broths was determined using the ABTS and FRAP methods (2.5 and 2.6 points, respectively), and the content of nucleotides and polyphenolic compounds was determined using the HPLC method (2.9 and 2.7 points, respectively).

### 3.11. Organoleptic Evaluation of Broths

A preliminary sensory evaluation was conducted to compare the basic broth with the broth enriched with mushroom extract (U+S). The assessment was performed by a panel of 10 non-expert participants. All samples were served at 80 °C and evaluated in triplicate. Participants assessed the taste, smell, appearance, and consistency of the broths on a 10-point hedonic scale with the following rating levels: 1—extremely undesirable, 2—very undesirable, 3—undesirable, 4—somewhat undesirable, 5—neither desirable nor undesirable, 6—somewhat desirable, 7—desirable, 8—very desirable, 9—extremely desirable, and 10—very, extremely desirable.

### 3.12. Statistical Analysis

All experiments were performed in triplicate, and the results were presented as the arithmetic mean ± standard deviation. The significance of differences between means was determined in ORIGIN, v.6.1 (Microcal, Northampton, MA, USA) using analysis of variance (one-way ANOVA) and *post hoc* Tukey’s test, with *p* ≤ 0.05.

## 4. Conclusions

The obtained results suggest that edible mushroom extracts are promising natural functional ingredients whose bioactive properties could potentially be effectively shaped through appropriate species selection and extraction methods. The *Agaricus bisporus* extract obtained using the combined method, in particular, demonstrated application potential, possibly enabling the improvement of the functional properties of zero-waste broths without substantially altering their basic sensory profile. However, due to the preliminary nature of the sensory evaluation, these observations should be interpreted with caution. Using the extract at a low concentration increased the product’s reduction potential and enriched its profile of non-volatile bioactive compounds, suggesting the feasibility of designing functional foods without the need to intensify umami flavor. These results open up prospects for further research on dosage optimization, extract stability during storage, and their effects in other complex food matrices, including products of mixed raw material origin and liquid and semi-liquid food systems. More broadly, the presented approach may align with current trends in sustainable food processing, demonstrating the real potential of mushroom extracts as a tool for valorizing by-products and developing innovative products with enhanced functional value.

## Figures and Tables

**Table 1:** Efficiency of extraction of biologically active compounds from *Pleurotus ostreatus* and *Agaricus bisporus*.

Extract	ssDNA [µg/mL]	dsDNA µg/mL	TPC [µgGAE/mL]	Antioxidant Activity Scavenging Free Radicals [%]	
				ABTS	DPPH
*P. ostreatus* U	320.00 ± 2.00 ^a^	480.67 ± 2.08 ^b^	43.34 ± 16.94 ^a^	49.18 ± 1.9 ^b^	28.67 ± 1.62 ^a^
*P. ostreatus* S+U	396.33 ± 1.53 ^b^	591.67 ± 1.53 ^c^	42.72 ± 8.52 ^a^	41.62 ± 1.4 ^a^	32.24 ± 2.01 ^a^
*A. bisporus* U	320.33 ± 0.58 ^a^	455.33 ± 0.58 ^a^	52.55 ± 9.99 ^a^	43.19 ± 1.7 ^a^	50.23 ± 0.92 ^b^
*A. bisporus* S+U	2696.00 ± 19.5 ^c^	924.33 ± 4.93 ^d^	97.08 ± 0.35 ^b^	60.15 ± 0.85 ^c^	48.76 ± 1.15 ^b^

TPC—total polyphenolic compounds as a gallic acid GAE, S—shaking, U—ultrasonic disintegration. The results are given as mean values from three experiments ± standard deviation; statistically significant differences were found between data designated with the different letters (a–d) in a given column (one-way ANOVA, *p* < 0.05).

**Table 2:** Concentration of polyphenols in mushroom extracts.

Extract	GAE	PCA	VA	CQA	CA	FA	QG
	µg/mL						
*P. ostreatus* U	54.15 ± 1.31 ^b^	98.72 ± 3.11 ^b^	0.18 ± 0.01 ^a^	0.01 ± 0.00 ^a^	8.16 ± 0.09 ^b^	0.49 ± 0.01 ^a^	0.88 ± 0.02 ^a^
*P. ostreatus* S+U	22.40 ± 0.25 ^a^	130.89 ± 4.12 ^c^	0.33 ± 0.01 ^b^	0.01 ± 0.00 ^a^	9.94 ± 0.08 ^c^	0.87 ± 0.01 ^b^	0.96 ± 0.01 ^a^
*A. bisporus* U	nd	nd	42.23 ± 0.98 ^d^	0.01 ± 0.00 ^a^	3.45 ± 0.01 ^a^	nd	nd
*A. bisporus* S+U	nd	14.19 ± 0.52 ^a^	31.20 ± 0.45 ^c^	nd	9.74 ± 0.12 ^c^	nd	18.98 ± 0.15 ^b^

GAE—gallic acid, PCA—protocatechuic acid, VA—vanillic acid, CQA—chlorogenic acid, CA—caffeic acid, FA—ferulic acid, QG—quercetin glucoside, nd—not detected. The results are given as mean values from three experiments ± standard deviation; statistically significant differences were found between data designated with the different letters (a–d) in a given column (one-way ANOVA, *p* < 0.05).

**Table 3:** Organic acids, sugars, and mannitol content in mushroom extracts.

Extract	MA	AA	LA	Glc	Mal	Mtl
	[µg/mL]					
*P. ostreatus* U	59.99 ± 1.11 ^a^	1743.36 ± 9.58 ^a^	nd	165.58 ± 3.11 ^c^	35.78 ± 1.12 ^d^	39.30 ± 0.59 ^a^
*P. ostreatus* S+U	85.15 ± 1.25 ^b^	nd	nd	201.26 ± 3.54 ^d^	22.35 ± 0.63 ^c^	37.84 ± 2.15 ^a^
*A. bisporus* U	665.89 ± 8.13 ^d^	1773.29 ± 8.11 ^a^	nd	81.23 ± 1.25 ^a^	7.65 ± 0.04 ^b^	1902.51 ± 5.21 ^c^
*A. bisporus* S+U	187.54 ± 2.32 ^c^	2182.76 ± 15.42 ^b^	2.61 ± 0.11 ^a^	125.95 ± 5.12 ^b^	3.57 ± 0.05 ^a^	1609.08 ± 3.49 ^b^

MA—malic acid, AA—acetic acid, LA—lactic acid, Glc—glucose, Mal—maltose, Mtl—mannitol, nd—not detected. The results are given as mean values from three experiments ± standard deviation; statistically significant differences were found between data designated with the different letters (a–d) in a given column (one-way ANOVA, *p* < 0.05).

**Table 4:** Nucleoside 5′-phosphate content in mushroom extracts.

Extract	5′CMP	5′UMP	5′GMP and 5′IMP	5′TMP	5′AMP	Other Nucleotides and Nucleosides
	[µg/mL]					
*P. ostreatus* U	14.68 ± 0.92 ^a^	nd	nd	9.09 ± 0.52 ^a^	nd	498.97 ± 3.54 ^a^
*P. ostreatus* S+U	14.52 ± 0.77 ^a^	nd	nd	9.82 ± 0.51 ^a^	nd	725.44 ± 5.14 ^c^
*A. bisporus* U	nd	9.66 ± 1.15 ^a^	nd	9.60 ± 0.72 ^a^	nd	580.04 ± 7.22 ^b^
*A. bisporus* S+U	nd	18.75 ± 0.78 ^b^	nd	10.93 ± 0.77 ^a^	nd	750.60 ± 8.15 ^d^

5′CMP—cytidine 5′-monophosphate; 5′UMP—uridine 5′-monophosphate; 5′GMP—guanosine 5′-monophosphate; 5′IMP—inosine 5′-monophosphate; 5′TMP—thymidine 5′-monophosphate; 5′AMP—adenosine 5′-monophosphate; nd—not detected. The results are given as mean values from three experiments ± standard deviation; statistically significant differences were found between data designated with the different letters (a–d) in a given column (one-way ANOVA, *p* < 0.05).

**Table 5:** Nucleotide content and antioxidant properties in control broth and mushroom extract-enriched broth.

Parameter	Control Broth	*A. bisporus*-Enriched Broth
Nucleotide content [µg/mL]		
5′CMP	20.53 ± 0.58 ^a^	19.98 ± 0.78 ^a^
5′UMP	nd	nd
5′GMP and 5′IMP	82.90 ± 1.33 ^a^	79.00 ± 1.19 ^a^
5′TMP	8.11 ± 0.54 ^a^	8.43 ± 0.73 ^a^
5′AMP	57.99 ± 0.98 ^a^	64.46 ± 0.55 ^b^
other nucleotides and nucleosides	970.09 ± 5.31 ^a^	1150.04 ± 20.15 ^b^
Polyphenol content [µg/mL] hydroxybenzoic acid derivatives hydroxycinnamic acid derivatives flavonoids Antioxidant activity	268.82 ± 1.52 ^a^ 18.45 ± 0.67 ^b^ nd	283.41 ± 3.15 ^b^ 14.34 ± 0.11 ^a^ nd
ABTS [mg TX/L]	511.2 ± 22.6 ^a^	507.44 ± 1.33 ^a^
FRAP [mg FeSO_4_/L]	614.5 ± 24.8 ^b^	801.85 ± 74.20 ^a^

5′CMP—cytidine 5′-monophosphate; 5′UMP—uridine 5′-monophosphate; 5′GMP—guanosine 5′-monophosphate; 5′IMP—inosine 5′-monophosphate; 5′TMP—thymidine 5′-monophosphate; 5′AMP—adenosine 5′-monophosphate; nd—not detected; FRAP—ferric reducing antioxidant power; the results are given as mean values from three experiments ± standard deviation; statistically significant differences were found between data designated with the different letters (a–b) in a given row (one-way ANOVA, *p* < 0.05).

## Data Availability

Data is contained within the article.
